# The use of household items to support online surgical knot-tying skills training: a mixed methods study

**DOI:** 10.1186/s12909-024-05549-1

**Published:** 2024-05-31

**Authors:** Sumayyah Ebrahim, Suman Mewa Kinoo, Maheshwar Naidoo, Jacqueline Marina Van Wyk

**Affiliations:** 1 Department of Surgery, Nelson R Mandela School of Medicine, College of Health Sciences, University of KwaZulu-Natal, Durban, South Africa; 2 Health Systems Research Unit, South African Medical Research Council, Cape Town, South Africa; 3 Department of Clinical and Professional Practice, Nelson R Mandela School of Medicine, College of Health Sciences, University of KwaZulu-Natal, Durban, South Africa; 4 Department of Health Sciences Education, University of Cape Town, Observatory, South Africa

**Keywords:** COVID-19, Surgical skills, Online teaching, Medical students

## Abstract

**Background:**

This study investigated the perceptions and performance of medical students regarding their engagement and learning of a knot-tying skill presented in an online demonstration format due to the emergency remote measures that accompanied COVID-19 restrictions.

**Methods:**

Final-year undergraduate medical students were invited to view an online demonstration of a one-handed knot-tying skill and practice the skill using common household items. They recorded their attempts and uploaded them onto the Flipgrid application. Completed attempts were scored using an adapted Objective Structured Assessment of Technical Skill (OSATS) validated tool. We used a mixed-methods sequential explanatory design; data regarding students’ engagement was gathered via a short questionnaire, and a Focus Group Discussion (FGD) was conducted to understand their learning experiences better. Descriptive statistics such as proportions and percentages were used to summarize categorical variables and median for continuous variables. Each video attempt was scored independently by two surgeons; reliability was determined using intraclass correlation; statistical tests were conducted at a 5% level of significance. Responses to open-ended survey questions and qualitative data from the FGDs were analysed using thematic analysis.

**Results:**

Seventy-one students participated in the exercise. Most students (91.5%) expressed confidence in their ability to perform the skill and were able to follow the steps in the video demonstration (83.1%). Median number of times needed to practice before video submission was 7.0 (Interquartile range [IQR] 5.0–10.0). Using the adapted OSATS tool; median scores on student attempts were 19.0 out of 21 (IQR: 17.0–20.0) for Assessor 1 and 18.0 out of 21 (IQR: 17.0–20.0) for Assessor 2, and overall scores showed good reliability between assessors based on intraclass correlation (0.86, 95% CI 0.79–0.90, *p* < 0.001). Qualitative insights from the students’ experiences in learning the skill were generally positive; it was a practical, experiential learning process and they valued the social aspects of learning via Flipgrid. Challenges expressed related to the need for in-person training and formal feedback on how to improve their technique. Suggestions to improve their learning included a request for an interactive session with immediate feedback on attempts, and being able to practice with a friend who would assist with videoing.

**Conclusion:**

Basic knot-tying can be taught with acceptable efficiency and student satisfaction using online methods with items available at home.

**Supplementary Information:**

The online version contains supplementary material available at 10.1186/s12909-024-05549-1.

## Background

During the COVID-19 pandemic all teaching and learning of final-year, undergraduate surgery students at a South African university was transitioned to a virtual teaching platform [1]. Virtual methods enabled the continuation of teaching and learning activities during this period when their access to the clinical surgical wards was compromised due to safety measures. There were however concerns regarding the clinical competency and preparedness of the students due to the lack of hands-on clinical and operative exposure [2–4]. Traditionally, surgical skills training follows an apprenticeship model in which experienced surgeons teach students one-on-one or in small groups in the operating theatre (OT) or a dedicated surgical skills laboratory [5]. Challenges with this model relate to the availability of instructors to deliver skills training due to their competing clinical commitments, and limited exposure of medical students to opportunities to assist in the OT due to the prioritized training of registrars and surgical fellows [5]. These are also similar challenges faced in our setting.

Video-based skills teaching with immediate or delayed feedback and assessment of skills using standardised tools were some of the main strategies used to teach surgical skills during the COVID-19 pandemic period. Surgical skills that could be practiced remotely were suturing and knot-tying skills and advantages included flexibility of in-home training, deliberate and repetitive practice [6, 7] with the potential for scalability [8], and decreased demands on faculty-teaching time [9]. Challenges related to quality of feedback especially if no feedback was provided on the skills performed [7, 10] and difficulty in evaluating psychomotor skill acquisition online [7, 8].

**Fig. 1:**
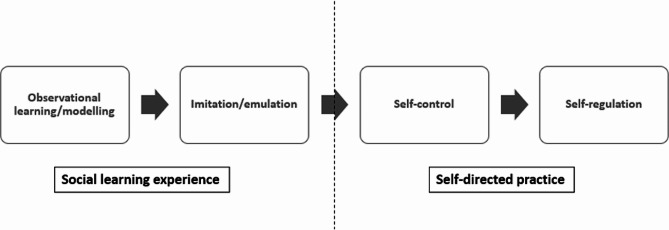
Social cognitive model of sequential skill acquisition [12]

Kolb (2014) described a “four-step cyclical process” of experiential learning comprising “thinking, feeling, watching and doing” [11]. The initial demonstration of a skill, via video or in-person, forms the basis of the theory of acquiring that particular skill [12]. The social cognitive model of sequential skill acquisition describes four phases of learning a psychomotor skill [13, 14]. The first two phases are considered as the social learning experience, which may lead to the last two phases i.e. self-directed practice of the skill (see Fig. 1) [12]. The phases include watching the skill being demonstrated by a proficient instructor in that skill (observation), followed by emulating the actions (imitation or emulation). This may lead to self-directed practice comprising self-control (“where students achieve automaticity in their behavioural technique”) and self-regulated performance of the skill (“where students learn to adapt their performance to changes in internal and external conditions”) 13, 14. 

Similarly, according to Ericsson’s theory of deliberate practice (1993) [15], expertise in a skill follows a process of breaking down a skill into its individual components and perfecting each step. Bandura’s social learning theory (1977) [16] describes learning as a cognitive process that takes place in a social context and through observation and self-reflection [6].

Routine student evaluations were conducted to identify aspects of the surgical online module at our medical school that worked well and those that students regarded as challenging [1]. Following the first round of engagement with the module, students who had received only online teaching perceived themselves as having gaps in the acquisition of their basic surgical skills. To address this immediate gap, an innovative online knot-tying skill exercise was introduced. This study explored the perceptions and performance of students of the knot-tying skill video demonstration, their self-paced practice of the skill during the surgical rotation, and their subsequent transfer of surgical skill to the authentic clinical setting.

## Methods

### Study design and participants

We used a mixed-methods sequential explanatory design [17] comprising an online questionnaire and focus group discussions (FGDs). This approach draws on an interpretivist paradigm to explore their experiences of the learning process [18]. Insights arising from quantitative data was complemented with further qualitative methods to clarify findings and provide a deeper narrative context [17]. Approximately 40–50 sixth-year medical students complete a 7-week rotation in an integrated General Surgery and Orthopaedics block. There were six such rotations between January to November 2022, with a total of 304 students. At the beginning of each rotation, students were invited to participate in an online one-handed knot-tying skills exercise via the Modular Object-Oriented Dynamic Learning Environment (Moodle) (https://moodle.com) learning management system. Students were informed about the purpose of the study and given the opportunity to participate using a Microsoft Forms (https://forms.office.com) sign-up sheet.

### Procedures

**Fig. 2:**
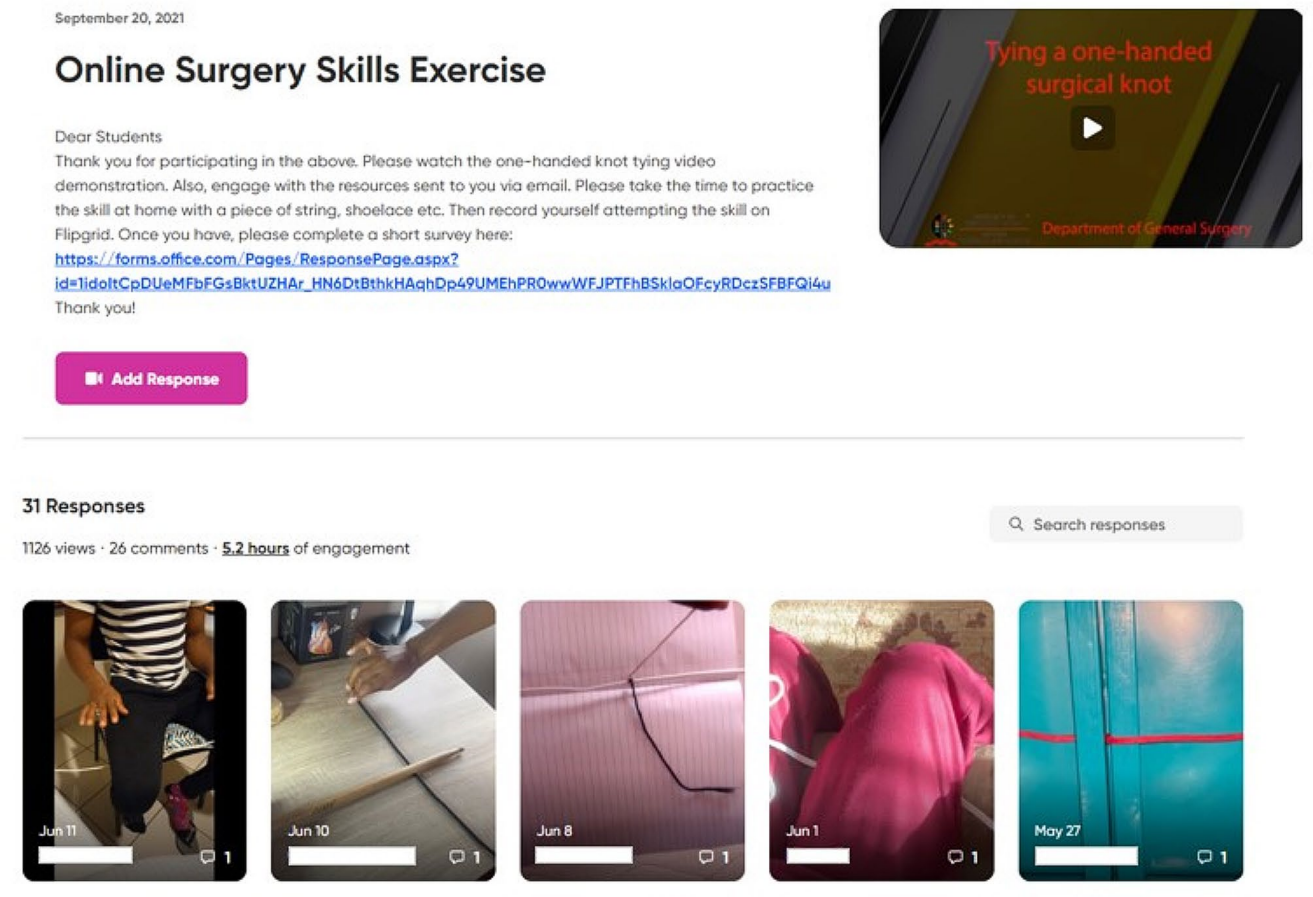
Flipgrid software interface

The Flipgrid software application (www.flipgrid.com) was used; the first author (SE) created a ‘grid’, to upload a video demonstration of the skill by an experienced surgeon and students were invited to upload their videos via a custom link (see Fig. 2). Flipgrid is a Microsoft online video discussion platform, that facilitates social learning between students [19]. It is available for free download, has an intuitive user interface and functions like many other video-based social media platforms (e.g., YouTube, Instagram and Snapchat) [19] and can be used on any type of device (computer, smartphone, or tablet).

In a synchronous Zoom (https://zoom.us) session, students who volunteered to participate in the exercise were briefed on the requirements together with a live demonstration of the skill. Students were invited to view the recording on Flipgrid together with related online content (final reef knot image, manual of steps, adapted Objective Structured Assessment of Technical Skill [OSATS] rubric) [20] and practice the skill at home using common household items such a string or shoelace on a door handle or the armrest of a chair. They were asked to record their attempts in a video response that had to be uploaded on Flipgrid for assessment. The completed attempts were then scored according to an adapted OSATS-validated tool (Table 1) [20], this tool differs from the original 1997 OSATS tool described by Martin et al. [21]. Formal feedback was not provided on their attempts. As soon as students posted their video attempts on Flipgrid, SE received an email notification of the submission which was viewed and ‘liked’ and a short comment was added on each video attempt on Flipgrid; such as ‘good attempt’, or ‘try again’ and students received an email notification of this. On average feedback was always provided within 24–48 h.

**Table 1:** Assessment of one-handed knot-tying techniques (Adapted OSATS scoring) [20]

	1 point	2 points	3 points
Tension of knot-tying cords during the procedure	Mostly loose	Mostly tight	Always tight
Motion	Many unnecessary moves	Less unnecessary moves	No unnecessary moves
Direction of the first knot	Wrong (Knot failure)	Not ideal (Knot failure)	Excellent
Direction of knots	Always the same	Partially alternating	Alternating
Stability of the knot	Loose loop, fell off the tying prop	The knot slipped off the tying prop	Good stability, knot did not slip off the tying prop
Finished knot	Not a reef knot	Loose	Perfect reef knot
Time	< 60 s	45–60 s	< 45 s

### Data collection

Students completed a short 20-item online questionnaire on the ease of using Flipgrid for learning, and their confidence in self-practice of the skill. The questionnaire was based on themes arising from the literature [5, 6] and piloted within the study team to ensure content validity and highlight any ambiguities that were corrected before implementation (see Additional file 1). A purposive sample - of students who submitted their knot-tying videos on Flipgrid and had completed the online questionnaire were invited to participate in an online FGD using the Zoom videoconferencing software [18]. The semi-structured FGD guide, Additional file 2, was developed by two authors (SE and JvW) in consultation with an experienced qualitative researcher (JLP). The guide comprised three sections that probed students’ experiences (learning and acquisition of skills), their challenges, and suggestions to improve the online skills training. Two facilitators were present at each FGD, one (SE) handled logistics related to the FGD, and the other (MM) led the FGD. At the last FGD, it was agreed that data saturation [18] had been reached and that inclusion of any more participants would not be necessary. A summary of the data collection methods is provided in Table 2.

**Table 2:** Summary of the data collection methods

Data collection strategy	Delivery	Description
Online survey	• Microsoft (USA) forms link provided on Flipgrid software and via email • Only one response was collected per student • To ensure anonymity, no email addresses or identifiers were collected	• Data was collected on demographics, ease of using Flipgrid, self-practice of the skill, challenges encountered, and suggestions for improvement • The questionnaire comprised dichotomous, multiple-choice, and Likert response scale questions with two open-ended questions
Focus group discussions (FGD)	• Three virtual, synchronous FGDs were conducted in 2022 • Participants were provided with study information sheets and consent forms before the FGD • Duration of the FGD ranged from 65–75 min and included a total of 20 participants	• FGD allowed for an in-depth exploration of student experiences, in a group setting of their peers • At the beginning of the FGD, there was a round of introductions, issues of confidentiality discussed, topic guide sections described, and verbal consent obtained for audio recording • The session was closed with a summary of the key topics, an opportunity for participants to make any final remarks, and information was provided on where they could access additional educational resources • Each FGD was audio recorded via Zoom and the online Zoom transcript was edited and reviewed for accuracy • Field notes were taken

### FGD team and reflexivity

The lead researcher, SE was present at all FGDs and is a lecturer and the module convenor for the integrated surgical rotation. She was responsible for inviting participants to the Zoom meetings, being available to provide information about the study, collating signed informed consent forms, and providing technical assistance during the session. She is known to the participants; thus, it was essential to have two facilitators per FGD. The second facilitator, MM a retired senior lecturer with a background in clinical skills teaching, led the FGDs; she was not known to the participants. Both facilitators were female, and participants included both males and females. Most of the participants knew each other as they were in the same clinical rotation at the time of the FGD.

### Data analysis

Statistical data analysis was conducted and results were presented in the form of descriptive and inferential statistics. Descriptive statistics such as proportions and percentages were used to summarize categorical variables and median for continuous variables. Each video attempt was scored independently by two assessors. Two methods were used to compare the scores of the assessors namely the intraclass correlation (ICC) and the non-parametric Wilcoxon test. Each video attempt was scored independently by two assessors; the degree of agreement between assessors and the correlation of scoring was determined using intraclass correlation coefficient (ICC). ICC describes how strongly units in the same group resemble each other. Statistical tests were conducted at 5% level of significance and 95% confidence intervals (CI) reported. Data analysis was conducted in R Statistical computing software (version 3.6.3 of the R Core Team) (https://www.r-project.org).

Qualitative data analysis was conducted using NVIVO 12 Pro software (https://help-nv.qsrinternational.com/12/win/v12.1.115-d3ea61/Content/welcome.htm) to organize, sort, and code the data. Thematic content analysis was conducted [22]. FGD questions guided the selection of the main themes of the data. Analysis followed a deductive process to develop a coding list based on the study objectives i.e. student perceptions of the online one-handed knot-tying exercise, including their experiences, challenges encountered, and suggestions. Similar codes were collected into subthemes, which were then grouped together under a main theme. The data was analysed and coded by one author (SE) and discussed with a second author (JvW), until consensus was reached.

### Ethics

This study was approved by the Biomedical Research Ethics Committee of UKZN (ref. no. BREC/00002686/2021). We obtained written informed consent from all participants. All data including a master list of the FGD participants, and their consent forms is stored electronically on password-protected computers. Participants were given the option to use a pseudonym [false name] if they preferred in the FGD. Names of participants were anonymised in the transcripts and subsequent qualitative data analysis. However, it is likely that as participants were known to each other, they may discuss the content of the session offline; none of the topics discussed were of a sensitive nature. We used the Mixed Methods Article Reporting Standards (MMARS) reporting guidelines to ensure comprehensive reporting [23].

## Results

Of the 304 enrolled students in 2022, 71 participated in the exercise (participation rate 23.4%). All 71 students submitted their video attempts on Flipgrid and completed the online questionnaire. The median (interquartile range [IQR]) age of participants was 23.0 (23.0–25.0) years, with more women 66.2% (47/71) than men 33.8% (24/71).

Most students were right-handed (88.7%, 63/71), and had no prior experience with the one-handed knot-tying skill (64.8%, 46/71). For the most part, students had the necessary equipment to perform the skill at home (71.8%, 51/71). 77.5% (55/71) used the Flipgrid application on their smartphones and the majority 91.5% (65/71) found the application easy to use and download. 83.1% (59/71) of students were able to follow the steps in the video demonstration, and 91.5% (65/71) expressed confidence in their ability to perform the skill. Median number of times needed to practice before video submission was 7.0 (5.0–10.0). Analysis of open-ended questionnaire responses identified challenges expressed by students regarding the asynchronous nature of learning the skill independently and the need for hands-on assistance and feedback. A video of one of the students’ attempts is available at https://youtube.com/shorts/B4ILty6Vl-E?%20feature=share.

**Fig. 3:**
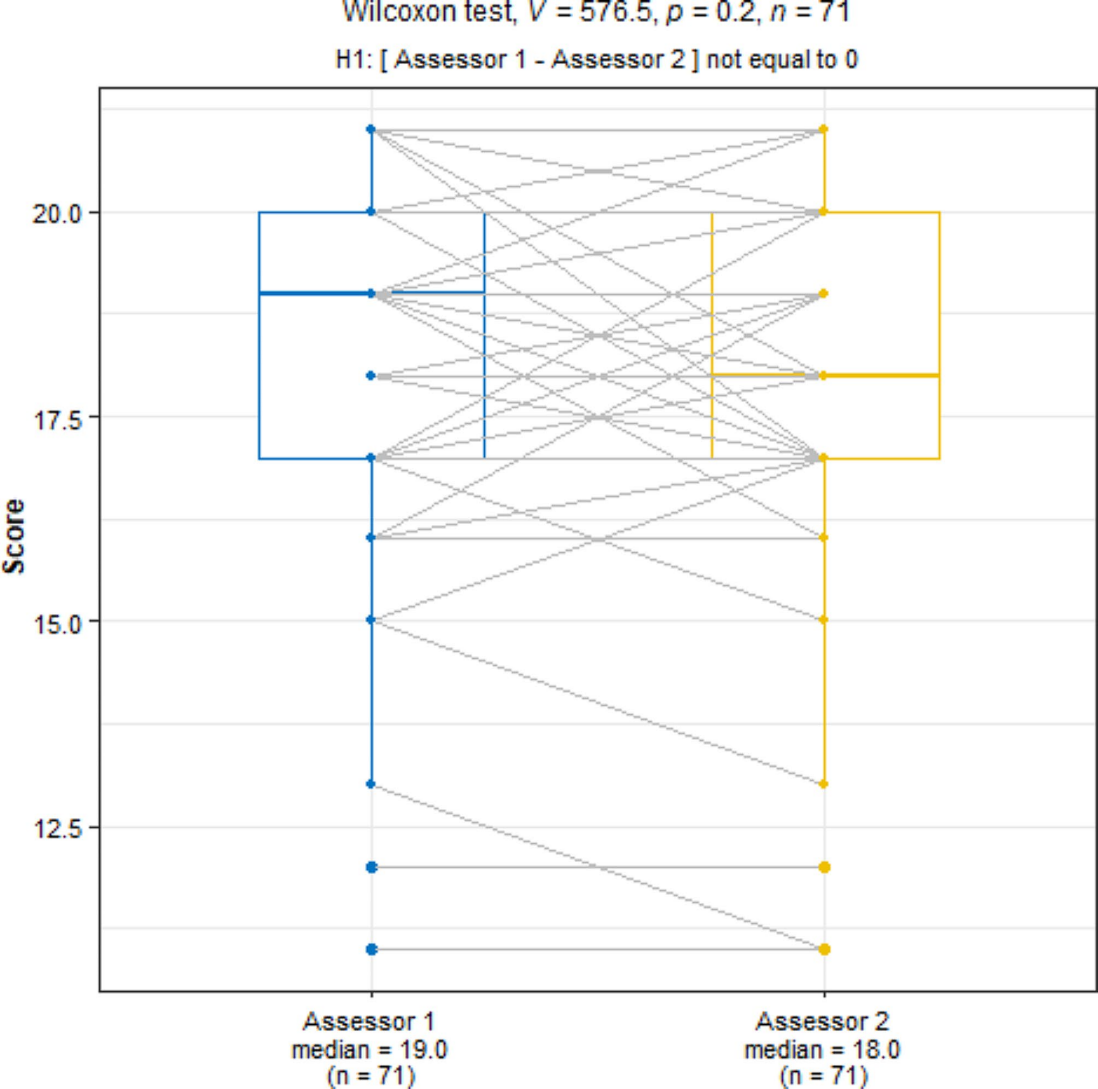
Comparison of participant scores between assessors

Using the adapted OSATS tool, scored out of 21 points; median scores obtained were 19.0 (17.0–20.0) for assessor 1 and 18.0 (17.0–20.0) for assessor 2. ICC values less than 0.5 indicate poor reliability, values between 0.5 and 0.75 moderate reliability, values between 0.75 and 0.9 good reliability, and values greater than 0.90 indicate excellent reliability [24]. The overall scores showed good reliability between assessors based on intraclass correlation (0.86, 95% CI 0.79–0.90, *p* < 0.001). The non-parametric Wilcoxon test, which assessed how comparable the scores of the same participants were between the two assessors, showed that the scores were similar (*p* = 0.200, Fig. 3).

**Fig. 4:**
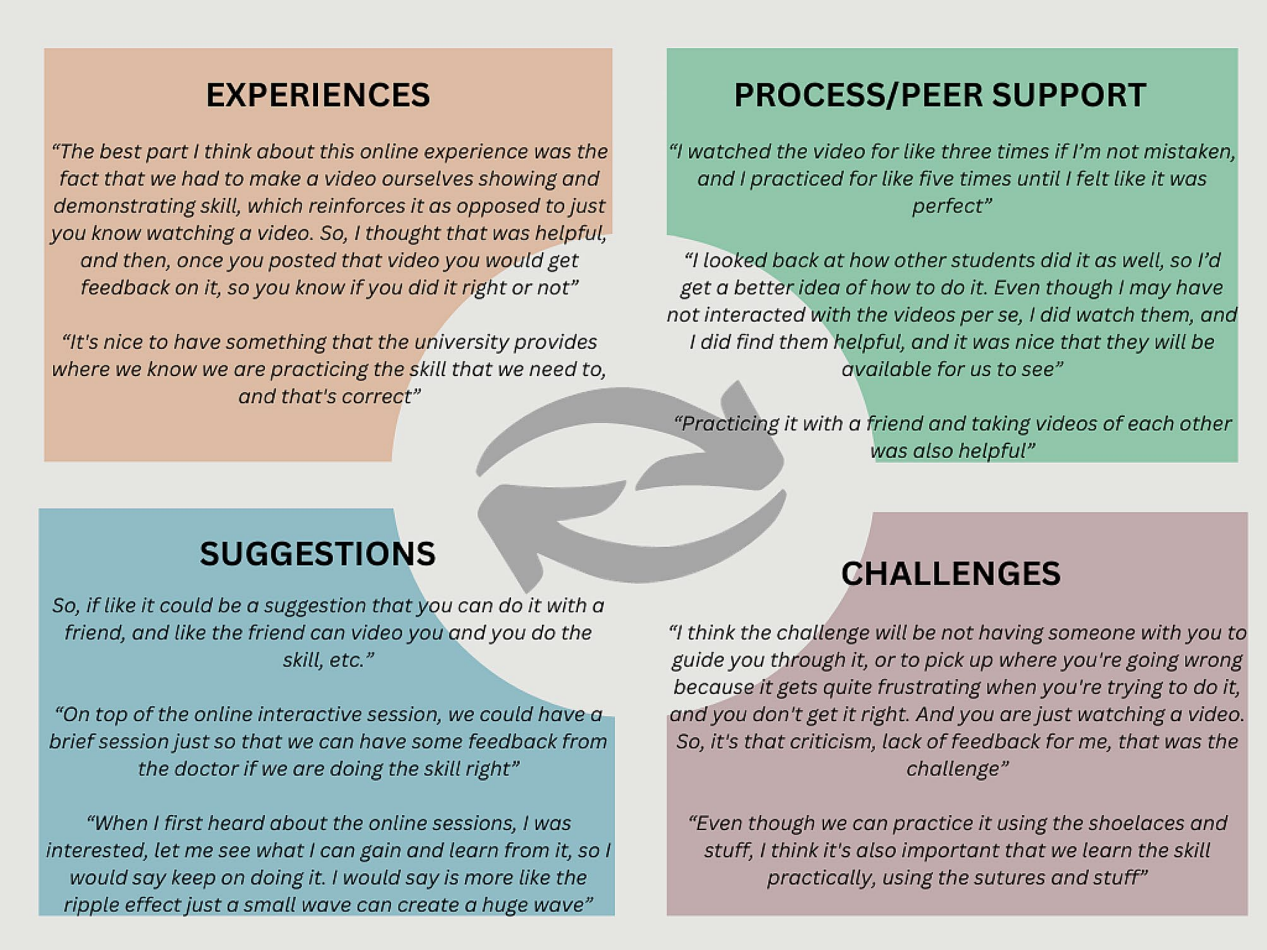
Major themes and feedback from FGDs

The major themes, subthemes, and representative quotes from qualitative data analysis of the FDGs are summarized in Fig. 4 and in Additional file 3.

### Experiences with online surgical skills training

Students reported positive learning experiences. For most, this was the first time they were engaging with online skills training; it was a practical, experiential learning process and they valued the social aspects of learning via the Flipgrid application. The self-paced activity, that was readily accessible and that could be practiced with items readily available at home were advantages highlighted by participants. None of the participants had the opportunity to practice the skill in the clinical setting but were willing to attempt it in a real-life setting if given the opportunity to do so.

### Challenges and concerns

The main challenge related to the lack of direct supervision and provision of formal feedback to individual students on their attempts. Other concerns expressed were on participants’ ability to execute the skill in a real-life, high-pressure setting with suture material and instruments etc. There were also challenges with videoing of the skill, positioning of the cell phone camera to obtain the correct angles and visibility of them performing the skill. Some students expressed difficulty in translating the two-dimensional (2-D) effect of the video to a 3-D application of the skill and expressed a need to visualize the skill from different video angles or positions. There were also concerns expressed with execution of the technical aspects of a skill, especially as these become more complex.

### Suggestions and recommendations

In the first FGD, there was a suggestion to pair up so that students could practice with a friend, do the skill together and video one another; this was implemented in the subsequent student groups. The need for supervision and feedback was a dominant view expressed by participants, especially in the last two FGDs conducted. Participants preferred learning the skill in a blended learning format, where they could practice the skill in a simulated setting such as in the skills lab, in group sessions under supervision, and reinforce the skill by watching the video content and practicing the skill on their own. Participants also reflected on the gaps in surgical skill training in the current curriculum and suggested that these be included earlier in their training.

## Discussion

This study describes an innovative approach to basic surgical skills training in a completely remote context using items readily available at home. Most students were able to follow the steps of the video skill demonstration and after a period of self-practice or practice with a peer, were able to successfully demonstrate and reproduce the final reef knot. Students expressed confidence in their ability to perform the skill and feedback from qualitative data showed that students had a positive learning experience. Challenges with the asynchronous nature of learning the skill related to a lack of direct supervision and formal feedback on their attempts. Findings were congruent between quantitative and qualitative data.

Surgical skill acquisition depends on both psychomotor proficiency and cognition and usually follows a traditional apprenticeship model with direct supervision and provision of immediate feedback [25]. The COVID-19 pandemic prompted the use of technology to facilitate skills training. On review of analytic studies, virtual skills training was shown to be non-inferior to face-to-face training [7, 10, 26–30]. Videos could be paused and played in slow motion for better visualisation of procedures [7]. However, it is challenging to evaluate psychomotor skill acquisition in the online space and this may be due to difficulties with obtaining an optimal camera position and the best angle in video-recorded attempts [7, 8, 31]. Quaranto et al. (2021) [8] described a novel first-person perspective of the instructor’s operative field using a cell phone camera mounted on a tripod to record knot-tying videos or use of a cell phone dock for positioning and videoing. This may be a strategy we could implement in the future to overcome some of the visuospatial challenges experienced by the students. Kumins et al. (2021) [5] developed a computer-based video training course on knot-tying and suturing skills introduced in discrete incremental steps for novice, pre-clinical medical students. Similar to our study, the training was self-directed, with minimal faculty involvement, and did not require a skills lab or one-on-one instruction. Students in that study showed significant improvement on post-course skills assessments and their self-assessment scores correlated well with faculty assessment scores, suggesting that they were able to self-monitor their performance and know when they had succeeded [5]. Furthermore, the concept of task-intrinsic feedback “where sensory-perceptual information that is a natural part of performing a skill” is valuable in self-directed video-based skills teaching and can provide important performance feedback [9]. This type of feedback relies on the auditory, visual, proprioceptive, and tactile pathways [9]. With respect to the visual pathway, examining the final product outcome can provide necessary feedback information to trainees; who probably know that they have performed well or poorly simply by looking at the final product [9].

Similar to our study, in two studies [7, 32], basic surgical skills were taught using items available at home such as shoelace or string for suture material, sewing needles, pliers or tweezers for instruments, and sponge or fruit peels to simulate a suture pad. Students achieved learning outcomes and reported high satisfaction [32]. In an Indonesian study [7], authors created a basic surgical knotting tutorial video and uploaded it onto YouTube. Students in the experimental group (*n* = 50), watched the video prior to completion of the first knotting skills task while students (*n* = 39) in the control group did not watch the video. All students submitted a video demonstrating the skill that was scored. Students in that study, similarly to our study, used items available at home i.e. shoelaces, and string to demonstrate the skill. This was followed by synchronous online Zoom classes for both groups where the skill was taught and individual feedback provided. Participants then submitted another demonstration video which was scored. The experimental group scored higher in the pre-test video compared to the control group, with a mean score of 10.850 versus 7.462, *p* = 0.000, however, post-test scores showed no significant difference between the two groups, *p* = 0.706. Thus, the authors found that the combination of asynchronous tutorial video and live skills demonstration was effective [7].

Strengths of this study include the incorporation of contemporary learning theories of experiential learning [11], deliberate practice [15], and social learning [16] in mastery of the one-handed knot-tying skill. Most students practiced the skill multiple times before submitting their videos. Flipgrid is a useful tool to facilitate social learning [19], it allowed students to view and interact with their peers’ attempts and motivated them to participate in the exercise. According to Bandura’s social learning theory, “new patterns of behaviour can be acquired through direct experience or by observing the behaviour of others” [16]. Students were able to practice without pressure at their own pace and had a reliable video source, sanctioned, and produced by the department to refer to. This is important to note as a major challenge with content posted on other platforms such as YouTube and other social media where there may be video quality issues and a lack of peer review and content control [33, 34]. Furthermore, knot-tying proficiency was evaluated in a blinded manner by two experienced surgeons using a validated tool and showed good interrater reliability.

Limitations of the study related to the lack of direct observation and formal feedback. Regarding direct observation, we did not have the necessary multiple camera and other equipment set up to facilitate one-on-one or even small group live skills teaching. Most students used their smartphones to record their attempts, without the use of tripods and other costly videoing equipment, and were issued with a set amount of internet data by the university for online learning. Furthermore, South Africa experiences an interruption of power supply to provide relief of demand on the electricity grid, thus maintaining an adequate and stable internet connection necessary to successfully conduct such sessions would have been difficult in the present study. Although informal feedback was provided to students on their attempts via Flipgrid, students did not feel that this was ideal and expressed a sense of frustration especially if they had practiced the skill on their own and had to try many times before obtaining the final reef knot. This could also be explained by the fact that most students were novices to this task with no prior experience. However, as described earlier, self-directed basic skills training was shown to be effective [5, 6], and examining the final product (completed reef knot) can provide valuable intrinsic performance feedback to students [9]. This may be an acceptable way to teach simple operative skills such as basic knot-tying or suturing, however, for more complex skills, external feedback from experienced instructors is necessary to inform skill performance and error detection [9]. The present study is a single-centre study with a relatively small sample size thus limiting the generalizability of quantitative findings. Poor uptake of the exercise by students may be explained by the fact that it was voluntary. SE was present at all FGDs; as module convenor, she is known to the participants, and this may have introduced response bias.

Reflecting on the strengths and limitations of the present study, as well as the suggestions by students, we have introduced basic knot-tying for fifth-year students in a simulated skills lab setting with small group instruction under the supervision of an experienced surgeon. Students now have access to the knot-tying videos created by the department and in this way, we can build on the gains of self-practice, convenience, and accessibility of resources offered by virtual methods in our setting.

## Conclusion

Basic knot-tying can be taught with acceptable efficiency and student satisfaction using online methods with items available at home. Video-based skills teaching is a cost-effective and scalable intervention that can be modified or adapted to teach other skills such as suturing, wound care, and biopsy techniques. However, further research is needed into the transferability and application of skills acquired virtually to the clinical setting and its effects on long-term skills retention.

### Electronic supplementary material

Below is the link to the electronic supplementary material.

Supplementary Material 1

Supplementary Material 2

Supplementary Material 3

## Data Availability

The datasets used and/or analysed during the current study are available from the corresponding author on reasonable request.

## Declarations

This study is part of a thesis submission by Sumayyah Ebrahim to the University of KwaZulu-Natal (UKZN) for the degree of Doctor of Philosophy (PhD Medicine).

## Abbreviations

OSATS: Objective Structured Assessment of Technical Skill
FGD: Focus Group Discussion
OT: Operating Theatre
CI: Confidence Interval ICC Intraclass Correlation Coefficient
UKZN: University of KwaZulu-Natal
BREC: Biomedical Research Ethics Committee
MMARS: Mixed Methods Article Reporting Standards
IQR: Interquartile Range 2-D two dimensional
