# Supplementary material for: The use of household items to support online surgical knot-tying skills training: a mixed methods study
Source: BMC Med Educ. 2024 May 31;24:605. doi: 10.1186/s12909-024-05549-1 (PMC11143630; doi:10.1186/s12909-024-05549-1)
Supplement: Supplementary file 1 — Supplementary Material 1 [file 12909_2024_5549_MOESM1_ESM.docx]

**Additional file 1. Surgical Skills Survey (One-handed knot tying exercise on Flipgrid)**

Good day, my name is Sumayyah Ebrahim from the School of Clinical Medicine, University of KwaZulu-Natal, [Ebrahims@ukzn.ac.za](mailto:Ebrahims@ukzn.ac.za)

I would like to invite you to take part in a survey to evaluate the online surgical skills exercise on Flipgrid. The questionnaire will take 10 minutes of your time and questions relate to your experiences during the skills exercise. Please do not start until you will have enough time to complete it in one go. Please close other programmes (e.g. chat or e-mail) to avoid distractions. By taking part, you are agreeing that you have read and understood the information about the study below. Please ensure you have read and understood this information before continuing.

***What is this project about, and do I have to take part?***

The findings from this study will be used to try to improve the online surgical teaching and learning programme at the institution.

***What are the benefits and risks of taking part?***

You may benefit from taking part in the survey by being motivated to look up information on online teaching during the COVID-19 pandemic. There are no foreseeable risks for you when taking part in the survey other than time spent on the survey and potential discomfort. Should you feel uncomfortable and want to leave the study you are free to do so without any consequences.

***What will be asked of you and what will happen to the information that you provide ?***

You will be asked questions about yourself, your past surgical skills training; and perceptions of Flipgrid software application use. Your data will be completely anonymous, and it will not be possible to identify you individually from your answers. This study has been ethically reviewed and approved by the UKZN and falls within the scope of a curriculum evaluation: Biomedical Research Ethics Committee (approval number: R201/04).

***How long will my data be stored for?***

Your anonymous data will be stored securely for up to 5 years by Sumayyah Ebrahim after the end of the study. At this point the data will be reviewed, and if they are still deemed to be of public interest, they may be retained for longer. If not, your data will be permanently deleted.

***Local Data Protection Privacy Notice***

Notice: The controller for this project will be Sumayyah Ebrahim. The data will be collected by Microsoft Forms.

This 'local' privacy notice sets out the information that applies to this particular study. Further information on how Microsoft Forms uses participant information can be found in the 'general' privacy notice: <https://support.microsoft.com/en-us/office/security-and-privacy-in-microsoft-forms-7e57f9ba-4aeb-4b1b-9e21-b75318532cd9>

***Concerns***

In the event of any problems or concerns/questions you may contact the researcher at Ebrahims@ukzn.ac.za or the UKZN Biomedical Research Ethics Committee, contact details as follows:

Biomedical Research Ethics Administration

Research Office, Westville Campus

Govan Mbeki Building

Private Bag X 54001

Durban, KwaZulu-Natal, South Africa

4000

Tel: 27 31 2604769 - Fax: 27 31 2604609

Email: [BREC@ukzn.ac.za](mailto:BREC@ukzn.ac.za)

***Consent***

I understand that:

- My participation is completely voluntary
- My data will be stored securely, however, no personal data will be stored, and my answer will be completely anonymous
- My data gathered in this study will be shared with relevant researchers
- Because I am submitting anonymous data, it will not be possible to withdraw my answers after they have been submitted

Please note that you can stop the survey at any time. This will *not* entail any penalty, and it will *not* affect your academic progress.

By ticking the box, you are agreeing that you are at least 18 years old, that you have read the information about the study, and that you voluntarily agree to participate.

I agree to participate in this study

I *do not* agree to participate in this study

Demographics

1. Please indicate your age:
2. Gender:

Woman

Man

Non-binary

Prefer not to say

1. I am currently based on the…

Central/Durban Hospital Platform

Decentralized/Stanger/Port Shepstone/Ngwelezane Hospital Platform

1. I did my Surgery Block in…

2021

2022

Regarding the use of Flipgrid,

1. I used the application on my:

Smartphone

Tablet

Laptop

Other ………………………………

1. I am familiar with Flipgrid and have used it before…

☐ Yes

☐ No

|  | **Strongly Agree** | **Agree** | **Neutral** | **Disagree** | **Strongly Disagree** |
| --- | --- | --- | --- | --- | --- |
| 1. Flipgrid was easy to download and use |  |  |  |  |  |
| 1. I was able to record the video for the knot-tying exercise easily using Flipgrid |  |  |  |  |  |

Regarding the knot-tying skill,

1. I had:

☐ No prior experience or knowledge of this skill

☐ Only theoretical knowledge of the skill

☐ Observed the skill before and practiced it

☐ Performed the skill before and could teach it

1. I am:

☐ Right-handed

☐ Left-handed

☐ Ambidextrous

1. Following the video demonstration of the skill, and before sending my own Flipgrid video, I practiced the skill on my own:

☐ Once

☐ Twice

☐ Three times

☐ Four times

☐ Five times

☐ Six times

☐ Seven times

☐ Eight times

☐ Nine times

☐ Ten times or more

|  | **Strongly Agree** | **Agree** | **Neutral** | **Disagree** | **Strongly Disagree** |
| --- | --- | --- | --- | --- | --- |
| 1. I had the necessary equipment/material to perform the skill at home |  |  |  |  |  |
| 1. I understood what I needed to do to perform the skill well |  |  |  |  |  |
| 1. I could follow the steps in the skills video demonstration |  |  |  |  |  |
| 1. I was able to see and hear the demonstrator on video clearly |  |  |  |  |  |
| 1. It helped that I could learn this on my own (and not all together with the group) |  |  |  |  |  |
| 1. I feel confident I will be able to do this skill by myself |  |  |  |  |  |
| 1. Learning surgical skills in this format makes me more interested in pursuing a career in Surgery |  |  |  |  |  |

1. I experienced the following difficulties/challenges with this type of video format, or with the asynchronous nature of learning it independently ……………………………………………………………………………………………………………………………………………………………………………………………………………………………………………………………………………………………………………………………………………………………………………………
2. I think that I would have learnt even better/more about knot-tying if you….………………….. …………………………………………………………………………………………………………………………………………………………………………………………………………………………………………………………………………………………………………………………………………………………………………………………………………………………………………………………………………………………………………………………………………………………………………………………
