# Supplementary material for: The use of household items to support online surgical knot-tying skills training: a mixed methods study
Source: BMC Med Educ. 2024 May 31;24:605. doi: 10.1186/s12909-024-05549-1 (PMC11143630; doi:10.1186/s12909-024-05549-1)
Supplement: Supplementary file 2 — Supplementary Material 2 [file 12909_2024_5549_MOESM2_ESM.docx]

**Additional file 2. Evaluating Student Perceptions of Online Surgical Skills Exercise on Flipgrid Focus Group Discussion Guide**

| **Date** | D | D | M | M | M | Y | Y |
| --- | --- | --- | --- | --- | --- | --- | --- |
| **Venue** |  | | | | | | |
| **Language** |  | | | | | | |
| **Facilitator ID** |  | | | | | | |
| **Note Taker ID** |  | | | | | | |
| **Number of Participants** |  | | | | | | |

**INTRODUCTION:**

- - Welcome participants and introduce yourself
  - Explain the general purpose of the discussion and why the participants were chosen
  - Explain the presence and purpose of recording equipment and introduce the note taker

**INSTRUCTIONS TO INTERVIEWER**

- - Interview schedule is targeted at students receiving online teaching
  - Before starting the FGD, the facilitator explains to the participants *(please state verbatim)*:
  - *We will switch the audio/video recorder on now*
  - *As you know from your informed consent, this discussion will be audio/video recorded today*
  - *Please verbally indicate that you are okay with this discussion being audio/video recorded*

**SECTION I: EXPERIENCE WITH ONE-HANDED KNOT-TYING EXERCISE**

| 1. **Tell me about your experience with this online surgical skill exercise.** *Probes: Please tell me what you liked or didn’t like about how you learn during this exercise* 2. **Have you tried to learn any other skills online before? Was this experience similar to or different from that? How so?** 3. **What was your preparation and process in attempting and executing the skill?** *Probes: Equipment needed, video quality and effects, Flipgrid software issues, self-practice, number of times video demo watched, time needed, peer support etc.* 4. **Were you able to apply this skill in the clinical setting?** *Probes: describe, on intake, in Operating Theatre, did you teach your peers the skill? etc.* |
| --- |

**SECTION II: CHALLENGES ENCOUNTERED**

| 1. **What are your concerns regarding learning skills via an online platform teaching and learning?** 2. **Ask each participant to name one challenge (not already mentioned) experienced with this asynchronous skill learning session.** *Probes: explain why you think that it did not work well* |
| --- |

**SECTION III: RECOMMENDATIONS/SUGGESTIONS**

| 1. **Give one suggestion (each) to improve the online surgical skills training?** *Probe: What changes would you suggest and why?* 2. **Would you like to try and learn another or other skills in a similar way?** *Probe:* *suggest one/2 you could learn in the online/ blended learning environment.* |
| --- |

**Is there anything else you would like to mention before we end the interview?**

**Thank you for participating in this discussion.**
