# Supplementary material for: The use of household items to support online surgical knot-tying skills training: a mixed methods study
Source: BMC Med Educ. 2024 May 31;24:605. doi: 10.1186/s12909-024-05549-1 (PMC11143630; doi:10.1186/s12909-024-05549-1)
Supplement: Supplementary file 3 — Supplementary Material 3 [file 12909_2024_5549_MOESM3_ESM.docx]

**Additional file 3. Summary of themes and sub-themes**

| **Themes** | **Sub-themes** | **Representative quotes** |
| --- | --- | --- |
| 1. **Experience** | Overall experience | - “So, for me this experience was quite pleasant, it was simple to do simple to follow it was quite enjoyable.” - “It was a positive experience, it was fun.” - “The best part I think about this online experience was the fact that we had to make a video ourselves showing and demonstrating skill, which really reinforces it as opposed to just you know watching a video.” |
|  | Accessibility and equipment | - “I particularly enjoyed the fact that we always had access to the videos, so you could be at hospital you could be quickly reviewing the videos. They were short videos. So that was nice and convenient, you could be in bed, and you could improvise. You could use anything that you had at home and try and re-do the skills and re-watch the videos. So, it was nice. You could take the skills away from just the skills lab, to wherever you are.” - “I also had quite a pleasant experience with all the resources that we were given as well as the software that was used. I also really enjoyed the fact that it was a video, so I could watch it multiple times and go back to certain steps, so it was easier to learn the knots in that way and the type of knots it was quite easy for any of us to video and learn it because we all we needed was string. Like we did not really need any other equipment, I thought that was quite nice.” - “I felt that it was very practical in the sense where we could refer to it as many times as we want, and as many occasions as we want, as opposed to if for example, we learned the skills in hospital then we must rely on memory, to learn that particular skill at that time. But with the online, I think we would refer to it, maybe later in the day, or even the next day, and that would be perfect.” |
|  | Self-paced practice | - “Well, I just thought that it was very convenient to be able to pause the video and watch exactly the part that you were struggling with, so you could watch a small part where you had difficulty, over and over again until you got it right, which made it more convenient than having to interact with someone and ask them to repeat over and over again. So, you really, just do the skill at your own pace, which was very helpful. We could take it wherever we went.” - “So, it's kind of nice to have a platform to learn at your own pace and to practice at home. I guess a lot of procedures are difficult to practice at home. But it would be nice to have a resource to refer to like a video.” |
|  | Preparation and process | - “For my preparation, I went through some of the resources that were prepared for us, as well as watching the video about five to 10 times. And then I tried practicing the knot by myself, without videoing and just seeing if I could get the technique correct and could remember what the video said and try to replicate that. I also went through some of the videos my colleagues did just to see what type of equipment they used to see if I had any of the stuff and could use the same thing. So, the only help that I needed was assistance for someone to help me video it so that I got a nice angle of me tying the knot, I just needed help videoing, but that’s it.” - “It was a good learning point, because I have never done it before, and was able to do the right thing. I mean, just watching from the video because the video was showing us all the steps, although I had to try it a lot of times. But I ended up achieving what I was supposed to do. So, I think it was a good experience.” - “I enjoyed the experience at first, I thought it was going to be extensive I thought that I was going to learn like a few skills, but then I realized that is actually one skill and the information that was given it was very helpful to me, even the marking rubric, we were given the marking rubric so I could just look at it and know what is expected of me and how I should do the knot and in terms of the Flipgrid app. It was very helpful, I even go back like a few weeks ago to check out what the other students did, and I liked some of the videos that I found very helpful to other students. The students were creative in whatever they did, they had good knots and I liked those videos. Yes, for me I enjoyed the experience, and I wish I had the exposure sooner, because I had a block before surgery, and I did not know how to tie a knot so yes that's my experience.” - “But the fact that it was recorded, and the guy did the video more than once I was able to watch a few seconds, then press pause, then, master the first step, then start the next step, press, pause, master the second step, and do it step by step, so that that's something that I really enjoyed. I thought the video really helped in terms of mastering step by step, as opposed to when you are learning real life where someone's just going to teach you the whole knot. And then you must just go do it.” |
|  | Flipgrid software | - “I didn't have any problems. It was quite straightforward from downloading the app, downloading the video on the app. Everything was just smooth sailing.” - “What was nice is the fact that we could also see previous students’ attempts, so, in addition to the video, we could look at other people that have attempted the knots and what their knots look like, before we did our own video as well. So, I feel like that also helped.” - “I haven't used any online material, that was the first time, using such an app for online learning. It was a bit difficult to knowing that we didn't have the necessary equipment but, overall, it was quite easy to use for me and I think that I could see more videos from other people that also motivated me to participate in the activity.” |
|  | Peer support | - “So, we were lucky that we practiced in a group so we could correct each other's technique. But if you're doing it solely the by yourself it might take long to learn the skill without any supervision” - “Practicing it with a friend and taking videos of each other was also helpful.” - “I also ended up teaching some of my classmates who didn't sign up for the for the skill, and they found it to be quite interesting as well, so they might also do it, maybe on their spare time” and another said that: “I can even do this skill now after like many weeks of not doing the skill.” |
| 1. **Challenges** | Need for supervision and feedback | - “I also think I learned best, when there is somebody there to supervise me and guide me. I'd like to think of myself as practical person. I love working with my hands, but I also need a little bit of guidance when I’m working with my hands just to make sure that what I'm doing it's correct and it's the right way of doing it.” - “I think the biggest challenge for me was the fact that I never received constructive criticism as I went along.” - “Sometimes I just find it better if I have someone who knows how to do the technique of the skill to advise me and give me actual feedback.” - “It is nice to actually have someone with you, like to give you that feedback like, and it's also easy to see in person how they are doing it, because it's sometimes difficult to visualize it from a video, if the angle is off, or it's not really explained clearly, but I think, I don't really have much concern when it comes to like online skills learning. I feel like some things can be learned online, and at the comfort, like at your own comfort, so that's an advantage, and I feel like that's kind of where the world is heading towards a lot of online things. But I still feel that it's a case for, like in-person skills training as well.” |
|  | Concerns about executing the skill in the clinical setting | - “I think my concern, similarly to what everyone has already tried to describe is that, even though I felt confident, by the end of this exercise with doing the skill with my equipment at home and a piece of string I was worried that it made me feel very different in actual clinical practice with suture material because suture material is quite thin compared to the string I was using whether my technique might not be so good” - “So, with the knot-tying as much as I've done it by myself, I haven't done it with the actual suture material in a patient, so I don't know if I’ll be able to successfully do it under pressure, or when it's slippery, because of the blood, etc.” |
|  | Challenges with videoing of their attempts | - “One of my challenges I had was that the actual videoing while you were doing the suture. So, I had to come up with weird ways; eventually I had to prop it up (cellphone) on a drawer, I got it but yes, videoing was one of the challenges.” - “The challenges I have were similar to what the previous students who just spoke, with the logistics of like setting up your camera making sure the angle was right, making sure that your fingers could be seen and so that the person who was assessing you could assess you well.” |
|  | Difficulty in translating the two-dimensional (2-D) effect of the video to a 3-D application of the skill | - “Perhaps it was the angles for me that made it a bit challenging for me to fully conceptualize what was happening” - The video was helpful, however, there is room for improvement, to make it easier for us to learn the skill. So, if you're actually watching someone do it in front of you, you have a 3-D perspective. It’s easier to learn a skill when you are actually watching someone doing it in front of you, than watching a video because it's like almost like you can't really see what’s happening in the video” |
| 1. **Suggestions** | Interactive online skills training with feedback | - “I was thinking for the first online session, it would maybe be an interactive session where the doctor is demonstrating while we watch, and we can ask questions on how he did the skill. I don’t like watching a video, and you don't know how we can ask, just asking each other and finding out. I think it would be better if the first session we could all just be in an interactive session with the doctor and can ask him how he did the certain skill, I think.” - “On top of the online interactive session, we could have a brief session just so that we can have some feedback from the doctor if we are doing the skill right.” - “I think for me the only suggestion I could make is that instead of submitting one video, you could do maybe 2 or 3 so that after you submit one, the evaluator could look at it and say maybe just put your hand like this, or you're doing this wrong; gives us some sort of feedback, and then we change it, and then we get the skill right you know. So maybe that will help students in the future.” |
|  | Practice with a friend | - “So, if like it could be a suggestion that you can actually do it with a friend and like the friend can actually video you and you do the skill, we can video them doing the skill.” - “However, let us be in a group setting where we practice thereafter and not individually at home. Because I feel like when you practice in a group setting you can learn off one another. So being able to learn from our colleagues, they might be able to teach us better that we teach us something we may not understand from the video, so I feel like in that way we learn from each other and be able to grasp the skill even quicker.” |
|  | Blended learning format, surgical skills training introduced earlier in the curriculum | - “Yes, we’re given an opportunity to learn the skill online, but then, if we could have like a feedback session where maybe during the online week, we could go, maybe to the Skills lab, and maybe demonstrate what we've learned from the online session to the consultant, or to the skills people who will be there, just so that we will be sure that we were doing the right thing.” - “If this was like an add-on, like an adjunct to learning the skill where, if we may be learned the skill in Skills lab, in-person and then had this video to constantly go back and like supplement; that I think a bit of both would obviously be perfect!” - “I think the online platform was perfect. It is very convenient easily accessible. It’s just the practical component where it needs to be in conjunction with the online. I agree with my colleagues that you kind of need in-person guidance before the online, it would be preferable if both the in-person and the online platforms can be accessible much earlier”. - “I feel there is a big gap within Medical School concerning suturing skills, because it's not really covered in our clinical skills in the first three years. So, I think it would be really effective if maybe this exercise was introduced to us earlier, maybe in fourth year because it is a skill that's quite, it's something that we as medical students can perform, and it would be quite helpful if we learned it quite earlier because we not only use it for surgery, which we can use it in other disciplines, as well as family medicine and so that was my recommendation. And then concerning other skills I’m also not quite sure because other skills may require more equipment and so I don't know if we can learn it solely online, but maybe doing that sort of blended learning, where we watch a video of a skill and then perform it with equipment provided may help.” |
|  | Preference to learn the skill in an authentic clinical setting | “I feel like also the confidence if I’m doing it in a clinical setting and the doctor is like, okay, you're doing it right, then that boosts my confidence, and I know the next time I’m going to do it, I’m going to be fine”. |
|  | Willingness to learn other skills in a similar way | - “The instrument suturing you can also do that. You can get tweezers I think anywhere in the shops and basically just try to use that and a piece of chicken that then just see how you can get it done.” - “I would like many other skills to be taught in this way, because I feel like it will improve our confidence levels when we go into the world. I feel like trying it for the first time on the human being; it's dangerous for them and for us, in terms of our anxiety and their lives.” |
